# Utilization of a healthcare failure mode and effects analysis to identify error sources in the preanalytical phase in two tertiary hospital laboratories

**DOI:** 10.11613/BM.2018.020713

**Published:** 2018-06-15

**Authors:** Adolfo Romero, Juan Gómez-Salgado, Adolfo Romero-Arana, Carlos Ruiz-Frutos

**Affiliations:** 1 Haematology Department, Hospital Universitario Virgen de la Victoria, Málaga, Spain; 2 Nursing Department, Health Sciences School, University of Málaga, Spain; 3 School of Nursing, University of Huelva, Huelva, Spain; 4 University Espiritu Santo, Guayaquil, Ecuador; 5 Fundación IMABIS, Málaga, Spain; 6 Environmental Sciences Department, University of Huelva, Huelva, Spain

**Keywords:** preanalytical phase, Healthcare Failure Mode and Effects Analysis, quality assessment, errors

## Abstract

**Introduction:**

The presence of errors in the preanalytical phase is a thoroughly studied problem. A strategy to increase their source detection might be the use of the Healthcare Failure Mode and Effects Analysis (HFMEA). The aim of this study is improving the capacity of identifying sources of error during the preanalytical period in samples provided by primary care clinics (PCC) with the use of the HFMEA as a tool in the laboratories of two tertiary hospitals.

**Materials and methods:**

A HFMEA was carried out in each laboratory, by means of the creation of groups of experts with similar characteristics (doctors and nurses from PCC and laboratory, support staff, and laboratory technicians). The Risk Priority Number (RPN) was calculated.

**Results:**

Items with elevated RPN were presented in both centers. The highest RPN were in LAB1: “two request notes for a patient” and “the segregation of oncology urgent samples” (both with 384), while in LAB2 was “the lack of information in patients with oral glucose overload test” (RPN 576). Considering the different steps in the preanalytical phase, LAB1 paid attention in sampling, samples reception and the programming in the Laboratory Information System, while LAB2 paid attention in the request form, the appointment system, sampling procedures, transport and reception.

**Conclusion:**

The laboratories prioritized the problems differently. However, both centers offer solutions to these possible sources of error. We proposed improvement actions that can be resolved easily, with a low cost for the system, mainly to schedule a specific formative programme and a deep revision of the existing protocols.

## Introduction

Although the presence of errors in the healthcare environment is a perfectly known risk both by patients and professionals, its existence implies the need to take into consideration actions for achieving the principle “*primus non nocere*” and avoiding harmful situations for the population (*1*).

In our country, the prevalence of adverse effects in primary care centers (PCC) was explained in the Patient Safety in Primary Health Care Study (in Spanish), which addressed patient safety in Spanish primary health care facilities and described that every 9.6 visits of 1000 will produce an adverse effect on the patient in PCC attention, although its degree of seriousness will be low (*2*).

On the other hand, the presence of errors in the preanalytical phase has been a widely studied topic in recent years. Several strategies have been developed as research lines, and actions have been set up in order to minimize the impact of these errors, studying their importance in different areas of the preanalytical phase in and out of the laboratory (*3*-*8*).

It is highly important to consider that the lack of detection or the misdetection of these errors can cause a potential risk, leading to misdiagnosis or wrong assessment in the patients’ follow-up. These errors could become serious, so their correct management is essential. Thus, the utilization of procedures that lead us to use diverse methodology is necessary, combining the detection of errors and the searching of the possible sources that may allow planning both preventive and corrective actions. In this sense, clinical laboratories have been characterized by an innovative profile that pursues the quality of their products introducing concepts such as quality control, quality assurance, or quality management (*9*).

Although the Failure Mode and Effects Analysis (FMEA) was first applied by the aerospace industry in the 1960s, and is usually employed in the analysis of a product or process, this method is valid for any kind of process or situation (*10*). Its adaptation to healthcare, Healthcare Failure Mode and Effect Analysis (HFMEA), was done by the US Veteran Health Administration and the Joint Commission on the Accreditation of Health Care at the end of the 1990s (*11*).

In this way, several studies have used the HFMEA as a useful tool to identify potential sources of errors and their possible causes and to take action to prevent them in the preanalytical phase with the previously stated aim (*12*, *13*).

In addition, although this aspect is not much developed in the literature, the role played by nurses and auxiliary staff is relevant, but they are not usually included in these studies. Despite this, it must be noted that nurses are in charge of the biological samples collection procedure, right in the heart of the preanalytical phase, and the auxiliary and support staff are key in its development. Several works have verified the importance of this role, insisting on the need of improvement in samples identification or in specific training, not always obtaining good results, although the relevance of this role has in fact been made evident as for the improvement of the patient safety (*14*-*16*).

The previously mentioned studies that used HFMEA have been performed in regional hospitals of our public healthcare system which have specific characteristics that could lead to a better control of the external factors such as the distance from the collection to the delivery point of samples (*10*, *11*).

In the current study, the results of two HFMEA are presented, developed in laboratories from our health regional system that receive samples from PCC and that are located at a greater distance from the sampling points. In addition, they include other considerations that might imply the relevance of a procedure of these characteristics.

Therefore, we hypothesized that obtaining data from those involved in the preanalytical phase could provide complete and useful information to develop improvement programs that can help enhance the detection of errors, decreasing their number.

Thus, the aim of this study is to improve the ability to identify error sources with the use of HFMEA as a tool during the preanalytical phase in samples from the PCC that arrive at the laboratories of two tertiary hospitals.

## Materials and methods

### Study design

We performed a descriptive study by means a HFMEA with the determination of the Risk Priority Number (RPN) of every item. The study was performed between April and October 2016.

### Participants

We included data obtained from two laboratories of the Andalusian public healthcare system: Virgen de la Victoria (Málaga, LAB1) and Juan Ramón Jiménez (Huelva, LAB2). Both centers receive samples from PCC to their areas, and both have a similar structure in preanalytical and samples reception areas.

LAB1 processes approximately 890,000 blood and urine samples yearly provided by PCC, and LAB2 receives 580,000 (data from 2015). Both laboratories receive samples from PCC located at a similar distance, use similar transportation and refrigeration means, and also have the same sample reception areas structure. The difference between samples is due to the different population of the areas (population in the province of Malaga is larger than in Huelva).

The first step was, by means of the analysis of the process of study and a brainstorm by our research team, to develop a scheme based on the preanalytical phase in which the different steps of the process where errors could appear were described. This scheme included six key areas that might be considered potential error sources in the preanalytical phase: analytical request and request form, appointment system, patient preparation, sampling, transport, and reception - preparation of samples. Then, we summoned the participants in order to proceed as has been previously described.

A group of professionals was set up, including two nurses (from PCC and laboratories), two laboratory technicians, two support staff and three doctors (2 analysts and 1 general practitioner), who were informed in detail of the purpose of the study and the methodology to use. Each group was coordinated by a member of the research team (in LAB1, the coordinator of the group was a clinical chemist, while in LAB2 the coordinator was a laboratory specialized nurse).

### Data presentation

Every participant was invited to comment on relevant aspects about the possible error sources they identified, following the order of the previously designed scheme, and they were asked to give a score from 1 to 10 for the severity rate (SR) and the occurrence rate (OR), being in both cases 10 the indicator of maximum rate, and to score the detection rate (DR), that values the capacity of being detected, with an inverse scale (higher probability of detection, lower numerical value). The RPN was calculated by multiplying the three data resulting (SR x OR x DR = RPN), then following a Pareto chart (*17*).

Once the possible errors were detected in the process, the participants were asked to offer solutions to the problems, opening a debate that sought the adoption of agreement proposals, which were finally noted down. We encouraged the participants to describe only error sources.

## Results

The analysis of the preanalytical phase, as we previously stated, revealed six sub-processes: analytical request and request form, appointment system, patient preparation, sampling, transport, and reception - preparation of samples. As it was described in the Materials and methods section, we encouraged the participants to describe only the error sources. So as to better understand the process, the area in which it is located, described above, is included between brackets next to each possible error source.

In LAB1, 24 potential errors were detected: 15 out of the laboratory facilities, and 9 within the same.

The highest RPN was 384 for two potential errors sources (existence of two request notes for a patient [analytical request and request form] and the segregation of urgent samples from the oncology service [reception - preparation of samples]). The most commented effect was the delay in processing samples or non-performance of the tests requested.

In LAB2, 16 potential errors were detected, 12 of them out of the laboratory facilities. The most commented aspect was the need to request a new sample. The highest RPN was 576, and it was given to the lack of information in patients with oral glucose overload test (patient preparation).

The actions proposed are focused on the revision of protocols, the improvement of the organization charts and the widening of information by means of establishing or improving communication ways and developing specific training programmes. All data are shown in Tables 1 and 2.

**Table 1:** Healthcare Failure Modal Effects Analysis data from Hospital Universitario Virgen de la Victoria Laboratory (LAB1)

Element / Function*	**Failure Mode**	**Effect**	**RPN**	**Proposed actions**
**Paper Analysis Request (*1*)**	Bad quality request form Incorrect completion Bar code identification not read	Sample not processed	60 96	Specific training Bar code identification on the back of the forms
**Analysis Request with Electronic form (*1*)**	Request not approved or authorised analysis Mistakes in request form	It might be done not approved or unauthorised analysis or correct test might be rejected	118	Information circuits improvement Correcting lack of information Revision of filters in Electronic form generation
**Laboratory Information System data input (*1*)**	Not activation of patient request form (bar code not read)	Sample not processed	32	
	Only in paper request form: two petitions one patient (double request form, one by General Practitioner, one by Clinical Specialist)	Sample not processed (overlap request)	384	Protocols dissemination improvement. Surveillance of sampling
	Lack of information or request not legible	Sample not processed	32	
	Mistake in patients demographic data	Sample not processed	32	
	Delay in scanning paper forms	Delay in testing	36	
**Appointment (*2*)**	Misinformation to patients (fasting time, medication, etc.)	Error in fasting time or any other preparation	108	Patient information improvement Preparation control in the Electronic form generation
	Error in urine samples which needs previous preparation	Bad or erroneous test	64	Preparation control in the Electronic form generation Containers delivery follow-up
**Sampling (*4*)**	Double utilization of labels	Electronic form: change bar code Paper request form: Sample rejected	6 18	Early detection
	Label/sample identification does not match	New sample	210	Potentially serious error. Extreme identification control
	Wrong test tube	Sample not processed. New sample	16	Special tests obtained only in Laboratory Sampling Room
	Bad quality in samples	Sample not processed (risk of misdiagnosis)	90	Improving training
	Incorrect patient identification	Sample processing error	180	Positive patient identification
**Transport (*5*)**	Delay	Bad quality of samples (new sample)	49	Redesigning collection and delivery routes
	Cold chain break	Bad quality of samples (new sample)	72	Extreme control of devices
	Loss of samples	New sample	16	
	Delivery at the wrong destination	Delay in samples processing Samples not processed	8	Increasing information
Element / Function*	**Failure Mode**	**Effect**	**RPN**	**Proposed actions**
**Samples receipt (*6*)**	Loss of sample tubes	Sample not processed	81	Improving delivery area control
	Bad centrifugation of samples	Samples not processed	81	Improving delivery area control
	Error in decantation or cold chain	Incomplete analysis or sample not processed	192	Coordination between delivery area and administrative section
**Aliquoting (*6*)**	Not enough samples	Not processed. Gives error MI	200	Information Coordination in/at AP
	Oncology urgent samples segregation	Delay in the reports generation	384	Protocols improvement and information enhancing
*Parts of preanalytical phase: (*1*) Analytical request and request form; (*2*) Appointment system; (*3*) Patient preparation; (*4*) Sampling; (5)Transport; (*6*) Reception and samples preparation.*RPN - Risk Priority Number.				

**Table 2:** Healthcare Failure Modal Effects Analysis data from Hospital Universitario Juan Ramón Jiménez (LAB2)

**Element / Function**	**Failure Mode**	**Effect**	RPN*	**Proposed actions**
**Analysis Request with Electronic form (*1*)**	Bad quality request form Incorrect completion Bar code identification not read	New sample	36	Check printers and bar codes Incidence notification
**Paper Analysis Request (*1*)**	Two patients with the same bar code It is no possible to read the form Relevant information not included (*i.e.* diuresis)	Possible incorrect result assignation Erroneous test scheduling Results will not be calculated	240 35 35	Redesign organisation Increase training and awareness (video, *etc*.)
**Patients preparation (*3*)**	Misinformation to patients (fasting time, medication, etc.), usually in appointments made by “Salud Responde”^†^	Altered results	360	Coordination between PCC, laboratories and “Salud Responde”^†^
**Sampling (*4*)**	No sample or inadequate tube Tube without bar code Bad sampling Lack of patient information (glucose overload test)	New sample Sample not processed Altered results	27 27 576	Check list made by the driver before the samples collection from the PCC Training Protocols revision and update
**Transport (*5*)**	Non-statutory Bridges Delay from sampling to laboratory more than 2 h	Bad quality samples Biological risk Possible altered results	486	Statutory Bridges with time and temperature control and secondary container Traceability sheets
**Samples receipt (*6*)**	Loss of tubes Bad centrifugation Delivery at the wrong destination	New sample Bad quality samples Not necessary or inadequate test made	81 8 245 192	Complete traceability follow-up Ensure the periodical revision in pre-analytical material (centrifuges, *etc*.) Training and protocols revision Specimen identification at origin
*Parts of preanalytical phase: (*1*) Analytical request and request form; (*2*) Appointment system; (*3*) Patient preparation; (*4*) Sampling; (*5*) Transport; (*6*) Reception and samples preparation. ^†^Telematic assistance given by the Andalusian Health Service, both by telephone and by electronic means. RPN - Risk Priority Number. PCC - primary care center.				

With reference to the content of the tables, there is a difference in the content of the ELEMENT / FUNCTION column between both aspects, as the data provided by the laboratories were not the same: the undetected problems were not reported and, subsequently, not included, and some results can be observed in one of the laboratories and not in the other one.

## Discussion

Risk management is an essential part of the quality culture in healthcare and necessarily implies a deep involvement of the professionals. In this sense, the HFMEA has proven to be, for years and in several fields, a reliable tool for the detection of possible error sources in structured systems, performing proactive risk management, an early identification of the causes of errors, and then, facilitating corrective actions (*18*, *19*). In this sense, the value of RPN helps prioritize procedures and strategies against the errors sources detected (*10*).

The results obtained in both centers have more similarities than differences, although it is necessary to specify that these differences may give greater value to the study due to the different characteristics of the corrective actions and the different approaches they require.

In particular, LAB1 considered as the most important errors those detected in the implementation of a new procedure (urgent oncology samples obtained in PCC) and the existence of duplicate requests, doubtlessly due to the novelty of the process and the communication errors detected in its implementation. In addition, LAB1 also considered factors such as the co-existence of general practitioner and specialist requests (both RPN 384) together with the lack of coincidence between the patients’ identification data and the labelled samples (RPN 210) and the existence of insufficient samples that prevent errors when aliquoting a sample (RPN 200). On its part, LAB2 highlighted the deficit of information given to the patient recipient of an oral overload of glucose (RPN 576), the delay in the transport of the samples to the laboratory (RPN 486), and the possible existence of the same code for two different patients (RPN 240). The rest of the data were very similar in both centers.

It is necessary to point out findings such as different RPN compared with other studies previously published made in our work environment (*12*). The reason might be that the participants declared high levels of error detection (that is, low detection rate), so this, doubtlessly, decreased the values of the parameter.

Although most error sources may be detected, a risk of misdetection still exists, as we previously commented. This situation forces us to plan strategies and actions that allow us to manage this problem as better as possible.

The data obtained from the HFMEA are very similar to those from the SWOT (Strengths, Weakness, Opportunities, and Threats) analysis previously performed by our team (*19*, *20*). Thus, we have to keep in mind that asking professionals has proved to be a useful practice, as could be seen when asked to give their opinions. This information provides enhancement strategies as it comes directly from those involved in the studied process. In this sense, the suggestions made by the laboratory staff were mainly focused on scheduling a specific training programme and also a deep revision of the existing protocols, which are relatively easy suggestions to perform. This is why we drafted a detailed report for the management team with instructions to carry out a detailed planning of activities in the near future.
